# Electrophysiological Dynamics of Visual-Tactile Temporal Order Perception in Early Deaf Adults

**DOI:** 10.3389/fnins.2020.544472

**Published:** 2020-09-23

**Authors:** Alexandra N. Scurry, Kudzai Chifamba, Fang Jiang

**Affiliations:** Department of Psychology, University of Nevada, Reno, Reno, NV, United States

**Keywords:** deafness, temporal processing, cross-modal plasticity, event-related potentials, multisensory perception, temporal order perception

## Abstract

Studies of compensatory plasticity in early deaf (ED) individuals have mainly focused on unisensory processing, and on spatial rather than temporal coding. However, precise discrimination of the temporal relationship between stimuli is imperative for successful perception of and interaction with the complex, multimodal environment. Although the properties of cross-modal temporal processing have been extensively studied in neurotypical populations, remarkably little is known about how the loss of one sense impacts the integrity of temporal interactions among the remaining senses. To understand how auditory deprivation affects multisensory temporal interactions, ED and age-matched normal hearing (NH) controls performed a visual-tactile temporal order judgment task in which visual and tactile stimuli were separated by varying stimulus onset asynchronies (SOAs) and subjects had to discern the leading stimulus. Participants performed the task while EEG data were recorded. Group averaged event-related potential waveforms were compared between groups in occipital and fronto-central electrodes. Despite similar temporal order sensitivities and performance accuracy, ED had larger visual P100 amplitudes for all SOA levels and larger tactile N140 amplitudes for the shortest asynchronous (± 30 ms) and synchronous SOA levels. The enhanced signal strength reflected in these components from ED adults are discussed in terms of compensatory recruitment of cortical areas for visual-tactile processing. In addition, ED adults had similar tactile P200 amplitudes as NH but longer P200 latencies suggesting reduced efficiency in later processing of tactile information. Overall, these results suggest that greater responses by ED for early processing of visual and tactile signals are likely critical for maintained performance in visual-tactile temporal order discrimination.

## Introduction

Natural timing discrepancies between multiple sensory signals inherently relay the source(s) and degree of congruency between those signals. Throughout development, with normal exposure to multisensory events, the brain develops an intrinsic strategy to compensate for the inherent differences in propagation and processing speeds of multimodal information allowing for coherent percepts (for review see Murray et al., 2016). This integrative mechanism is largely driven by sensitivities to the temporal and serial nature of the particular sensory cues. For instance, due to the fact that visual information typically precedes auditory information, individuals are more sensitive to temporal asynchronies for auditory-leading compared to visual-leading information (Conrey and Pisoni, 2006; van Eijk et al., 2008; Cecere et al., 2016). This is also reflected in the asymmetry of the temporal binding windows (Conrey and Pisoni, 2006; van Wassenhove et al., 2007; Powers et al., 2009; Hillock et al., 2011; Stevenson et al., 2012), the period of time within which multiple stimuli are likely to be perceptually integrated, indicating that exposure to patterns of natural temporal delays within multimodal signals is a major driver in fine-tuning this sensitive process. Temporal recalibration of audiovisual (Fujisaki et al., 2004) and visual-tactile (Hanson et al., 2008) stimuli (i.e., the perceptual shift in perceived simultaneity of auditory and visual signals following repeated exposure to a consistent temporal delay between the two cues) emphasizes the flexibility of this integrative process across modalities. Such permeability is crucial for adapting to different external environments and maintaining temporal congruency and subsequent integration across sensory systems. However, absence of sensory input during development may significantly alter temporal discrimination and decoding, particularly if the deficient modality inherently conveys temporal and sequential information (i.e., the auditory system, for review see Conway et al., 2009). Indeed, early deaf adults demonstrated reduced sensitivity for sensory-motor timing and deficits in sensory-motor temporal recalibration for visual stimuli in the central visual field suggesting impairments in perception of sensorimotor causality (Vercillo and Jiang, 2017).

Recalibration of temporal order perception is thought to reflect the brain’s interpretation of external signals rather than the physical asynchrony between signals. This notion is supported by findings from auditory-induced cueing of a visual temporal order judgment task where attention toward one of two visual signals (left or right) was induced via an auditory signal prior to either the synchronous or asynchronous presentation of the two visual cues. Participants demonstrated a clear perceptual bias toward the visual signal from the cued location as being presented first, regardless of simultaneous presentation and any latency differences in early visual evoked components (i.e., P100) suggesting that such a perception is not driven by increased visual processing speed (McDonald et al., 2005). However, increased amplitude of the visual P100 did accompany this condition, theorized to reflect enhanced signal strength of the cued visual signal that is interpreted as temporal primacy during later stages of processing (McDonald et al., 2005). Intriguingly, during asynchronous trials, the latency of the early visual P100 component was approximate to the veridical delay between the two visual signals, regardless of participant’s perception (McDonald et al., 2005). Activation and connectivity patterns between regions of the prefrontal cortex, insula, and superior temporal sulcus (STS) are likely responsible for higher order processing of both the physical temporal order dynamics of the stimulus pair and the perceptual state of the participants (Noesselt et al., 2012). As the STS is inherently multisensory, absence of a modality induces reorganization of sensory inputs to and connections between primary sensory cortices and this multimodal STS region (Meredith and Lomber, 2011; Meredith et al., 2011) which should subsequently affect temporal order processing.

Auditory input does appear to play a particularly important role in creating refined resolution for temporal processing. The Auditory Scaffolding Hypothesis suggests that early auditory experience provides a necessary framework, or scaffold, to develop sensitivity to temporal information, including serial order, since these properties are fundamental to sound (Conway et al., 2009). In early deaf (ED) adults, tactile duration, but not spatial, discrimination was impaired compared to normal hearing (NH) controls (Bolognini et al., 2011). Compared to spatial discrimination, ED adults also show degraded temporal discriminatory abilities whereas NH did not show different sensitivities between spatial and temporal tasks (Papagno et al., 2016). In a complex temporal bisection task, ED adults demonstrated impaired performance that was eliminated when spatial cues were linked to the temporal differences between stimuli (Amadeo et al., 2019). Performance in these spatially varied temporal bisection tasks did not vary among NH individuals suggesting that early deafness exerts limitations on precise and independent development of temporal processing (Amadeo et al., 2019). Deficits were also found for unisensory visual and tactile simultaneity judgments in ED compared to NH adults (Heming and Brown, 2005) suggesting impaired temporal processing due to early auditory deprivation. Additionally, children with cochlear implants showed deficits in serial learning of visual and auditory information (for review see Pisoni et al., 2016) providing further support for the need of early auditory experience to precisely discriminate serial information of sensory cues.

Alternatively, some studies don’t show any deficits in visual or tactile temporal processing abilities of ED individuals and suggest that compensatory mechanisms lead to recruitment of auditory areas by intact modalities enabling normal or even enhanced perceptual abilities. For instance, tactual discrimination thresholds, estimated using stimuli ranging from 2 and 300 Hz, and tactile temporal order discrimination thresholds, estimated from a task discriminating which of two vibrotactile stimuli was presented first, did not significantly differ between ED and NH (Moallem et al., 2010). Similarly, visual temporal order thresholds did not differ between ED and NH, although ED adults had faster response times than NH during a visual temporal order discrimination task (Nava et al., 2008). These findings support unaltered temporal processing in unimodal contexts for early deaf adults.

The conflicting results outlined above were found while assessing unisensory temporal processing abilities, however, as temporal discrepancies between signals significantly affects integrative processes, alterations in multisensory temporal processing are expected in ED adults. The extent of facilitation from audio-tactile simultaneous presentation compared to unimodal presentation was examined in both congenitally deaf cochlear implant (CI) users and late deaf CI users (age of onset 7 years or later) by comparing reaction times for a bimodal stimulus to reaction times for unimodal stimuli (Nava et al., 2014). While both CI groups showed evidence of audio-tactile interaction, measured as multisensory facilitation (i.e., faster reaction time for bimodal compared to unimodal stimuli), only congenital CI users had weaker redundancy gains compared to their age-matched NH controls. Further, there was a significant correlation found in the congenital CI group, not the late deaf CI group, that showed faster tactile reaction times were associated with weaker redundancy gain. Overall, this may suggest that early deafness results in enhanced reliance on the tactile modality, possibly from cross-modal reorganization that strengthens and increases inputs for tactile information. A similar conclusion was found in a recent study that compared evoked neural dynamics of unisensory visual and tactile stimuli to synchronous visuo-tactile stimulation. The latency of the tactile N200 component (defined as the negative peak within 152 – 252 ms after stimulus presentation) was modulated by simultaneous presentation of a visual stimulus in NH only, not ED, suggesting limited multisensory interactions and diminished visual influence over tactile processing in ED (Hauthal et al., 2015). This finding also reflected behavioral results which showed deficits in the extent of multisensory facilitation in ED adults compared to NH (Hauthal et al., 2015). Taken together, this may suggest that ED individuals assign higher reliability to tactile information which would limit the visual system’s influence over tactile processing and behavioral redundancy effects would be reduced in the presence of a hyper-salient tactile cue.

Another study that supports the notion of absent early auditory experience modifying multisensory processing and degraded visual influence over somatosensation showed that ED adults had increased susceptibility to a tactile induced double flash illusion compared to NH (Karns et al., 2012). In addition, the strength of the illusion was positively associated with somatosensory activation in primary auditory cortex (PAC) of ED (Karns et al., 2012). The increased likelihood of integrating asynchronous stimuli, as predicted by PAC activity during tactile stimulation, further suggests that the tactile modality primarily drives the integration of asynchronous stimuli underlying these illusory percepts in ED more so than NH. Interestingly, opposing findings were reported in a group of CI users that were tested with an audio-induced double vibration illusion. Only NH participants perceived illusory tactile stimuli when multiple auditory cues were presented, indicative of auditory-tactile interaction in NH but not in CI users (Landry et al., 2013). This finding described CI users that had congenital deafness and CI users that had progressive deafness (onset between 7 to 17 years of age), suggesting that a lack of auditory exposure, regardless of the time period, affects multisensory interactions even following CI implantation (Landry et al., 2013).

Presumably, for multisensory interactions in this auditory-to-tactile direction to occur (not in the tactile-to-auditory direction as show by Karns et al., 2012), early auditory experience is required. These conflicting findings may be indicative of unequal modulations on the remaining modalities as a consequence of absent early auditory experience. In other words, the tactile system of ED individuals seems to exert a greater cross-modal influence than the visual or partially restored auditory system (in the case of CI users). Similar to differential neural dynamics found during a simultaneity judgment task in normal hearing individuals exposed to auditory-leading versus visual-leading stimulus pairs (Cecere et al., 2016), it is likely that different mechanisms drive multisensory binding depending on the leading sensory input and that these mechanisms are differently affected by early sensory experience.

By examining how stimuli from one modality (i.e., visual) modulates the processing of a subsequent stimulus from a different modality (i.e., tactile), effects of early auditory deprivation on the multisensory integration process can be better understood. As precise integration relies on efficient decoding of temporal information between signals, what is the consequence of auditory deprivation on cross-modal influence of temporally disparate signals? Using a visual-tactile temporal order judgment task, this project investigated how information from one modality (i.e., visual) affected the processing of temporally disparate lagging signals from the opposite modality (i.e., tactile) in ED compared to NH. In line with previously reported findings, we would expect reduced influence by leading visual stimuli on tactile processing in ED compared to NH but similar influence on visual processing by leading tactile cues for both groups (Hauthal et al., 2015). When a significant cross-modal influence is exerted on sensory processing of the subsequent stimulus in the pair, we would predict reduced amplitudes of the ERP component (i.e., reduced visual P100 amplitudes in NH compared to ED for tactile-leading visual SOA conditions). As ED have demonstrated larger amplitudes for visual and tactile processing during unisensory detection tasks (Hauthal et al., 2015), we also would expect greater amplitudes in the ED group for the synchronous condition across ROIs. If efficiency of sensory processing is reduced (or enhanced) by early deafness for either visual or tactile modality, we would predict slower (or faster) latencies of the respective ERP components (McDonald et al., 2005). In addition, following the auditory scaffolding hypothesis, we would expect less precise multisensory temporal processes, manifested in worse performance accuracy during the TOJ task by the ED group. This prediction is further supported by previously reported impairments in multisensory interactions for congenital CI users (Nava et al., 2014) and ED (Hauthal et al., 2015) compared to NH. To investigate effects of auditory deprivation on processing multimodal signals, ERP components reflecting sensory processing were compared. Specifically, the influence of a leading stimulus on the early and late components of a subsequent stimulus were investigated across different SOAs between ED and NH within occipital and fronto-central electrodes. Finally, spatial topography differences in early and late stages of sensory processing were examined for both groups.

## Materials and Methods

### Participants

12 early deaf with bilateral, severe to profound hearing loss (M = 41.73 ± 8.45; 5 males; cause and age of deafness onset reported in Table 1) and 12 age- and sex-matched normal hearing controls participated in this study. All participants were right-handed and reported normal or corrected-to-normal vision. Participants were screened for any history of neurological or psychiatric disorders, history of brain injury, antipsychotic medications and cognitive decline. Participants provided signed informed consent before any experimentation. All experiment protocols were reviewed and approved by the Institutional Review Board at the University of Nevada, Reno.

**TABLE 1:** Demographic information on ED subjects.

**ID**	**Age (years)**	**Handedness**	**Clinical description**	**Age at deafness onset (months)**	**Auditory deprivation (left; right) (dB)**
ED1	46–50	R	Hereditary	Birth	90; 90
ED2	40–45	R	Spinal meningitis	9	90; 90
ED3	50–55	R	Unknown	18	105; 110
ED4	40–45	R	Spinal meningitis	4	100; 100
ED5	30–35	R	Hereditary	15	Total; 85
ED6	50–55	R	Unknown	Birth	85; 90–100
ED7	40–45	R	Maternal gestational measles	Birth	100; 90
ED8	50–55	R	Hereditary	Birth	90; 90
ED9	35–40	R	Cytomegalovirus	12	Total; 90
ED10	30–35	R	Unknown	Birth	80; 80
ED11	30–35	R	Unknown	16	120; 120
ED12	36–40	R	Spinal meningitis	18	110; 110

### Stimuli

The visual stimulus was a 33 ms white circle of 3.5° centered around a fixation cross, presented via the Psychophysics Toolbox using a Display + + system with a refresh rate of 120 Hz (Cambridge Research Systems, Rochester, United Kingdom). The 50 ms tactile stimulus of 50 Hz was generated using the PiezoTac tactor device (Engineering Acoustic, Casselberry, FL, United States). To approximate the same central location as the visual stimulus, the tactile stimulus was always presented to the tip of the participant’s right index finger positioned directly below the center of the display.

### Experimental Paradigm

Throughout each experimental block, a white fixation cross was presented in the center of the screen on a gray background. During each trial, a visual and tactile stimulus were presented at varying stimulus onset asynchronies (SOAs) where negative SOAs represent tactile-leading conditions and positive SOAs represent visual-leading conditions. Based on pilot data, 7 SOAs were chosen so that 2 were outside of the average TBW (± 250 ms), 2 were within the average TBW (± 30 ms), 2 were at the limit of the average TBW (± 100 ms), and the final SOA of 0 ms was a simultaneous, control condition. Each SOA was repeated 60 times, in a randomized order, for a total of 420 trials separated into 3 experimental blocks.

After the visual-tactile pair was presented, participants were asked to make a temporal order judgment (TOJ) about the 2 signals by pressing “1” on the keyboard for a flash first response and “2” for a touch first response using their non-dominant left hand. To reduce muscle artifacts into the cortical signal, participants waited to enter their response until 800 ms after the second stimulus presentation, indicated when the fixation turned green. Trials were separated by a variable interval between 1000 – 1300 ms.

### Behavioral Analysis

Accuracy of temporal order judgments were quantified for all asynchronous conditions. For each individual, the average correct response was calculated for each asynchronous SOA level tested and individual proportions were averaged together across participants within both the NH and ED groups. Individual’s proportion of ‘visual first’ responses were also plotted as a function of SOA value and fit with a cumulative gaussian function. The mean and the standard deviation were estimated from the cumulative distribution as estimates of sensitivity or just noticeable difference (JND) and perceived synchrony or point of subjective equality (PSE), respectively (Weber, 1834; Fechner, 1860; Burr et al., 2009; Scurry et al., 2019). The JND represented the smallest temporal difference between visual and tactile signals that an individual could detect while the PSE represented the perceptual bias of a participant’s perception of visual-tactile synchrony. Individual JND and PSE values were averaged across participants within each group.

### Electroencephalography Data Acquisition and Analysis

Participants performed the visual-tactile TOJ task while EEG data were continuously recorded from a 128 channel BioSemi Active 2 system (BioSemi, Amsterdam, The Netherlands). In addition to the standard 10–20 electrode locations, this system included intermediate positions. Default electrode labels were renamed to approximate the more conventional 10–20 system (see Supplementary Figure S1 in Rossion et al., 2015). 4 additional channels recorded electrooculography (EOG) signals, two channels on the lateral sides of each eye to detect horizontal movement and two channels above and below the right eye to detect vertical movement (i.e., blinks). EEG was sampled at a rate of 512 Hz and processed offline using EEGLAB (v.14_0_0b) and ERPLAB (v.6.1.3) with MATLAB R2013b (Mathworks, Natick, MA, United States).

First, EEG data were bandpass filtered from 0.1 to 40 Hz with a second order, non-causal Butterworth filter and re-referenced to the common average reference. Channels were identified for rejection using the TrimOutlier plugin (v.0.17) based on a threshold of ±200 μV. Across participants, an average of 2.8 (± 4.24) channels were rejected and spherically interpolated. Next, epochs of 1200 ms, beginning 200 ms before trial onset (defined as onset of the first stimulus in the visual-tactile pair), were extracted from continuous data. Epochs corrupted by artifacts were identified following visual inspection and an average of 9.00 (± 7.78) trials (<2.2%) were rejected across participants. Blink and eye movement artifacts were corrected in the epoched data using Independent Component Analysis (ICA). Event related potentials (ERPs) were calculated for each individual as the average of all epochs within each experimental condition. ERPs were baseline corrected relative to the mean amplitude of the pre-trial interval of 200 ms. ERPs were then averaged across participants within the NH and the ED groups.

To quantify the electrophysiological dynamics of processing a sensory stimulus preceded by a stimulus from a different modality, amplitudes and latencies were extracted for the lagging stimulus of the asynchronous experimental conditions for each participant. Amplitudes were defined as the maxima peak within a pre-defined time window while latencies were estimated as the time to peak onset within the time window. Specifically, amplitudes and latencies of early (P100) visual components were estimated in the 120 – 180 ms window post visual onset, based on Basharat et al., 2018; Setti et al., 2014. To maintain consistency across SOA level, the window shifted based on the SOA (positive SOAs and 0 SOA: 120 – 180 ms; −30 SOA: 150 – 210 ms; −100 SOA: 220 – 280 ms; −250 SOA: 370 – 430 ms). Visual components were examined within a visual region of interest (ROI), defined as the average of ERPs from 12 occipital channels (I1, POI1, O1, POO5, POOz, Oz, OIz, Iz, I2, POI2, O2, POO6) (Setti et al., 2011). A later visual component (N200) was not included after initial analysis showed extremely variable and inconsistent amplitude values across participants for all SOA levels. Amplitudes and latencies of the early (N140) and late (P200) tactile processing components were extracted from time windows defined as 100 – 180 ms and 190 – 250 ms post tactile onset, respectively (Hauthal et al., 2015, 2013). Again, to retain consistency and continuity of the overall group trends, these windows shifted based on the SOA for both tactile N140 components (negative SOAs and 0 SOA: 100 – 180 ms; + 30 SOA: 130 – 210 ms; + 100 SOA: 200 – 280 ms; + 250 SOA: 350 – 430 ms) and tactile P200 components (negative SOAs and 0 SOA: 190 – 250 ms; + 30 SOA: 220 – 280 ms; + 100 SOA: 290 – 350 ms; + 250 SOA: 440 – 500 ms). These estimates were done within a Fronto-Central (FC) ROI made up of the average of 8 channels (Cz, C1h, C2h, FCC1h, FCC2h, FCC1, FCC2, and FCz) and known to reflect somatosensory processing (Ito et al., 2014; Hauthal et al., 2015).

To quantify topographic differences between groups for each SOA level and component, an index known as the global dissimilarity measure (DISS) (Lehmann and Skrandies, 1980) was computed for the same windows used to examine the respective ERP component at the respective SOA level. DISS was estimated as the square root of the mean squared difference between scalp potentials of each electrode which were normalized by their instantaneous global field power (GFP) (Murray et al., 2008). GFP was calculated as the standard deviation of the whole scalp electric field (Murray et al., 2008). DISS provides a topographic index between 0 and 2 where 0 represents homogeneity and 2 represents inversion of the scalp topography (Murray et al., 2008).

### Statistical Analysis

As ROIs had unequal number of channels, separate mixed ANOVAs were calculated for each region of interest using the between factor of group (NH vs ED) and the within factor of SOA (7 levels). Due to multiple ANOVAs to investigate differences in both amplitude and latency of visual P100 in occipital, tactile N140 in FC and tactile P200 in FC, the critical alpha level used to determine statistically significant effects will be 0.0167 (0.05/3). As processing of simultaneous visual-tactile events was an additional aspect of investigation, separate independent *t*-tests with a Bonferroni corrected alpha value of 0.0167 (0.05/3) were used to examine differences between ED and NH groups during the 0 ms SOA condition in tactile N140 and tactile P200 components within FC and in the visual P100 component in occipital ROI. Independent *t*-tests were also used to compare PSE and JND values between groups as well as for an *a priori* comparison of components evoked during synchronous presentation of visual-tactile stimuli.

Non-parametric permutation tests were used to quantify the significance of estimated DISS values for each component at the respective SOA levels. Following the commonly used topographic ANOVA (TANOVA) method (Murray et al., 2008), individual subjects were randomly assigned to either the ED or the NH group and new group-averaged ERPs were computed. Then, new DISS values were estimated for each SOA at each of the components as reported in section 2.5. This procedure was repeated for 2500 iterations for each ERP component at each respective SOA level and empirical distributions were generated. If the original DISS estimates fell within an *a priori* defined significance level of 0.05, they were deemed significant.

All statistical analysis was performed in R statistical software.

## Results

### ED and NH Adults Had Equivalent Performance Accuracy, Temporal Order Sensitivity and Perceived Synchrony

**FIGURE 1:**
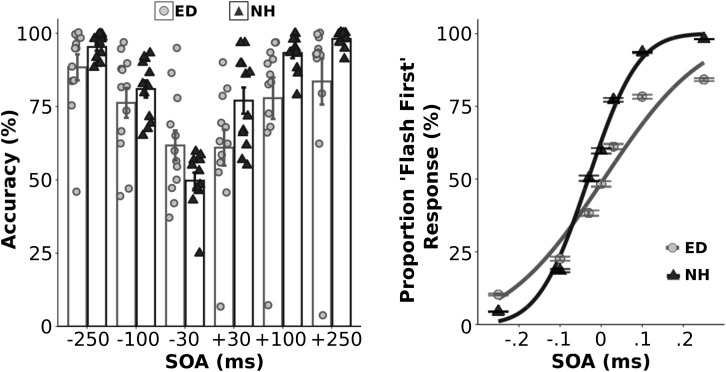
Behavioral performance in the visual-tactile temporal order judgment task. **Left panel:** Group averaged and individual data for behavioral accuracy are plotted for ED (light gray boxes with light gray circles) and NH (black boxes with black triangles) groups for each asynchronous SOA test level. **Right panel:** Average proportion of flash first response at each SOA level along with the fitted cumulative normal distribution is plotted for ED (light gray circles w/light gray line) and NH (black triangles w/black line). **Error bars reflect standard error.

Initially, we quantified the proportion of correct responses for each asynchronous SOA condition within each group (Figure 1, left panel). As expected, a mixed ANOVA showed an effect of SOA [F(5,110) = 26.57, *p* < 0.001, *n_*p*_^2^* = 0.55] on performance accuracy. Although ED and NH groups did not perform differently overall [F(1,22) = 3.03, *p* = 0.10], there was a significant interaction between group and SOA [F(5,110) = 3.85, *p* < 0.01, *n_*p*_^2^* = 0.15]. However, follow up *t*-tests that compared group accuracy performance at each SOA level using a corrected p value of 0.0083 showed that ED did not perform significantly different from NH at any SOA (uncorrected *ps* ≥0.047). Average psychometric functions from both groups are displayed in the right panel of Figure 1. Two separate independent *t*-tests also revealed that ED and NH groups did not differ in their sensitivity (JND) [t(22) = −0.27, *p* = 0.79] or point of subjective equality (PSE) [t(22) = 1.69, *p* = 0.11] for the visual-tactile TOJ task.

### Visual and Tactile Components Induced by Synchronous Visual-Tactile Stimulation

**FIGURE 2:**
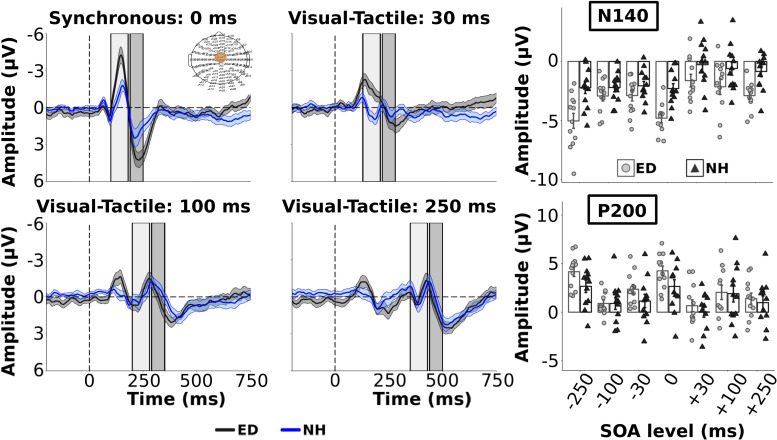
Group average ERPs and tactile components from Fronto-Central ROI. Group averaged ERP waveforms from FC electrodes (ROI shown in **top left panel**) are plotted for ED (gray solid line) and NH (blue dashed line) groups for synchronous and 3 visual-leading conditions. The gray and blue shaded envelopes around the waveforms correspond to the ± SE for the ED and NH group-averaged waveform, respectively. Amplitudes of the tactile N140 and P200 components are shown **(right column)** for group-averaged and individual data from ED (light gray boxes with light gray circles) and NH (black boxes with black triangles) groups extracted from the respective time windows (N140: light gray box; P200: darker gray box) displayed in the ERP plots.

**FIGURE 3:**
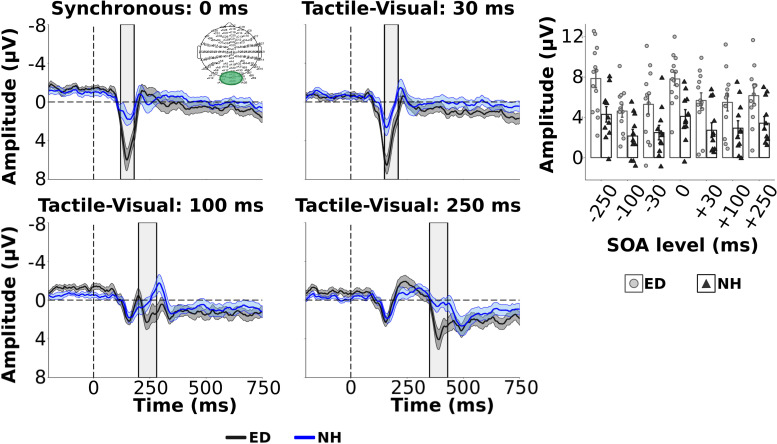
Group average ERPs and visual components from occipital ROI. Group averaged ERP waveforms averaged from electrodes within occipital electrodes (ROI displayed in **top left panel**) are displayed for ED (dark gray solid line) and NH (blue dashed line) groups for synchronous and 3 tactile-leading visual conditions. The gray and blue shaded envelopes around the waveforms correspond to the ± SE for the ED and NH group-averaged waveform, respectively. Amplitudes of the visual P100 component are shown for group-averaged and individual data from ED (light gray boxes with light gray circles) and NH (black boxes with black triangles) groups extracted from the post-stimulus time window relative to the visual cue (shown by light gray box on ERP plots) for each SOA level **(top right panel)**.

As we were interested in differences in the electrophysiological dynamics of simultaneous visual-tactile events between ED and NH adults, *a priori* independent *t*-tests with Bonferroni correction (0.05/3 = 0.017) compared the amplitudes and latencies of tactile N140 and P200 in FC ROI and the visual P100 component within the occipital ROI. Amplitudes of the tactile N140 component were significantly larger in ED compared to NH in FC ROI [t(22) = −3.51, *p* < 0.01, *d* = 1.43] while amplitudes of the tactile P200 component were comparable between the two groups [t(22) = 1.88, *p* = 0.07] (see Figure 2 top left panel). In addition, there was no significant difference between ED and NH latencies of tactile N140 [t(22) = −1.10, *p* = 0.28] or tactile P200 components [t(22) = 0.84, *p* = 0.41] in FC ROI. In occipital ROI, ED adults had a significantly larger amplitude for the visual P100 component [t(22) = 2.90, *p* < 0.01, *d* = 1.19] (see Figure 3, top left panel) while there was no group difference for visual P100 latency estimates [t(22) = −0.33, *p* = 0.74].

### Visual Influence on Early and Late Tactile Sensory Processing Components

Group averaged ERPs are shown in Figure 2 for the 3 visual leading conditions (positive SOAs), and synchronous condition for comparison, for ED (dark gray line) and NH (dark blue line) in FC ROI. The 3 tactile leading conditions were not plotted in the FC ROI as we wanted to demonstrate the change in the somatosensory ERP induced by a preceding visual stimulus. For group average tactile N140 amplitude and latency values across all SOAs, see Supplementary Table S1. As observed in the top right panel of Figure 2, the amplitudes of the early tactile N140 component were significantly larger for ED compared to NH [F(1,22) = 11.5, *p* < 0.01, *n_*p*_^2^* = 0.34] and there was a significant effect of SOA [F(6,132) = 15.62, *p* < 0.001, *n_*p*_^2^* = 0.42]. However, these were qualified by a significant interaction [F(6,132) = 2.41, *p* < 0.05, *n_*p*_^2^* = 0.10]. Follow up pair-wise comparisons with Bonferroni corrected alpha value of 0.007 (0.05/7) were performed for each SOA to determine which conditions had amplitude differences between ED and NH. For the synchronous and smallest SOA levels (±30), ED had significantly larger tactile N140 amplitudes than NH [*t’*s(22) < −3.51, *p’s* < 0.001, *d’*s ≥ 1.43]. However, there was no group difference at ±100 or ±250 SOAs [*t’*s(22) > −1.77, *p’*s > 0.09].

While there was no group difference in tactile N140 latencies [F(1,22) = 0.01, *p* = 0.93], there was an effect of SOA [F(6, 132) = 4.31, *p* < 0.001, *n_*p*_^2^* = 0.16] and a significant interaction [F(6,132) = 2.53, *p* < 0.05, *n_*p*_^2^* = 0.10]. To explore this interaction *post hoc*, separate *t*-tests were performed for each SOA level. Only at −250 and −100 SOAs did ED have significantly shorter latencies than NH [*t’*s(22) < −2.3, corrected *p’*s < 0.05, *d’*s ≥ 0.94]; there was no latency difference between groups at the other 5 SOA levels [*t’*s(22) < 1.71, *p’*s > 0.10].

For the amplitudes of the tactile P200 component within FC ROI, there was no significant difference between ED and NH groups [F(1,22) = 1.13, *p* = 0.30] nor a significant interaction between group and SOA [F(6,132) = 1.74, *p* = 0.12]. However, there was a significant effect of SOA [F(6,132) = 23.5, *p* < 0.001, *n_*p*_^2^* = 0.52] (see Figure 2, bottom right panel) with follow up comparisons showing that the amplitude of the synchronous and −30 SOAs were significantly larger than the +30, ±100, and −250 SOAs (corrected *p’*s < 0.002). Further, the +100 SOA had a significantly smaller amplitude than the +250, −30 and −250 SOAs (corrected *p’*s < 0.01, *d’*s ≥0.75) but not than the +30 or −100 SOAs (*p* > 0.28). P200 amplitudes did not significantly differ between +30 and +250 (*p* = 1.0) (see Supplementary Table S2 for group average tactile P200 amplitude and latency values).

ED adults had significantly longer tactile P200 latencies than NH [F(1,22) = 4.90, *p* < 0.05, *n_*p*_^2^* = 0.18]. In addition, there was a significant effect of SOA [F(6, 132) = 3.22, *p* < 0.01, *n_*p*_^2^* = 0.13] but no significant interaction. Bonferroni corrected pairwise comparisons revealed that the latency in the + 250 SOA was significantly longer than +30, −100 and −250 SOAs (corrected *p’*s < 0.04, *d’*s ≥ 0.81). No other comparisons were significant (corrected *p’*s > 0.06).

### Tactile Influence on Visual Sensory Processing Components

Next, we quantified the influence of tactile information on the processing of subsequent visual signals, within occipital ROI, presented at variable delays. While we were more interested in how the tactile stimulus may affect subsequent processing of the visual stimulus, Figure 3 shows the group averaged ERPs for ED and NH adults across the synchronous and tactile-leading visual conditions (3 negative SOA levels). Supplementary Table S3 reports group averaged visual P100 amplitude and latencies values for all SOAs. A mixed ANOVA showed that ED group had significantly larger visual P100 amplitudes than NH group [F(1,22) = 10.07, uncorrected *p* < 0.01, *n_*p*_^2^* = 0.31]. While there was no significant interaction [F(6,132) = 0.65, uncorrected *p* = 0.69], SOA level did significantly affect visual P100 amplitudes [F(6,132) = 12.28, uncorrected *p* < 0.001, *n_*p*_^2^* = 0.36]. *Post hoc* comparisons with Bonferroni correction revealed that the visual P100 amplitude induced by the synchronous condition (0 ms) was significantly larger than ± 100 and ± 250 SOAs (corrected *p’*s < 0.01, *d’*s > 1.03) but not ±30 SOAs (corrected *p’*s ≥ 0.30). As expected, amplitudes did not differ between the three visual-leading tactile conditions (+SOAs) (corrected *p’*s > 0.19). However, the visual P100 amplitude was significantly larger for the −30 SOA than both −100 and −250 ms SOAs (corrected *p’*s < 0.001, *d’*s > 1.39).

There was no significant difference between visual P100 latencies estimated from occipital region for ED and NH groups [F(1,22) = 0.33, *p* = 0.57] nor was there a significant interaction [F(6,132) = 1.65, *p* = 0.14]. However, there was a significant effect of SOA [F(6,132) = 5.18, *p* < 0.001, *n_*p*_^2^* = 0.19] with follow up pairwise comparisons using Bonferroni correction revealing that the latency in the −30 SOA condition was significantly shorter than the synchronous (corrected *p* < 0.001, *d* = 1.23) and −100 SOA (corrected *p* < 0.05, *d* = 0.78) but not −250 SOA (corrected *p* = 0.26) nor any of the positive, visual-leading SOAs (corrected *p* > 0.09).

### Widespread Distribution of Activity During Visual-Tactile Processing in ED

**FIGURE 4:**
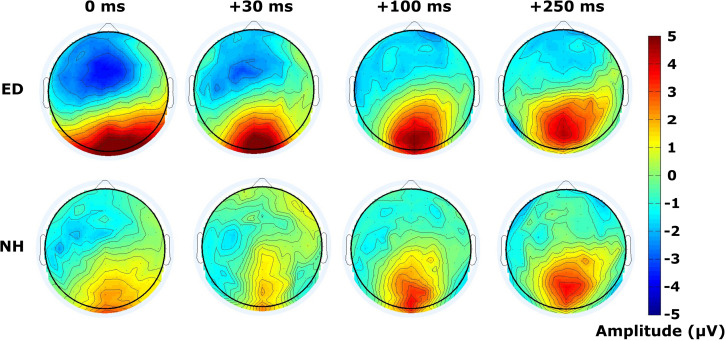
Scalp topography of mean amplitudes for tactile N140 component. Scalp topographies of mean amplitudes within time window designating the tactile N140 component are displayed for ED **(top row)** and NH **(bottom row)** groups, for synchronous and 3 visual-leading conditions (positive SOAs).

**FIGURE 5:**
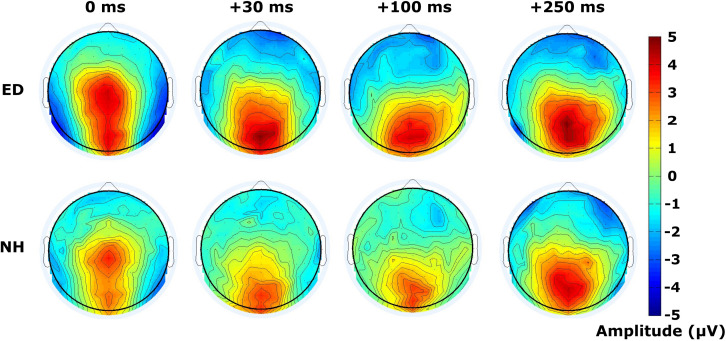
Distribution of activity within tactile P200 time window. Scalp topographies of mean amplitudes within time window designating the tactile P200 component are displayed for ED **(top row)** and NH **(bottom row)** groups, for synchronous and 3 visual-leading conditions (positive SOAs).

Scalp topographies are displayed in Figure 4 for both ED (top row) and NH (bottom row) groups for the tactile N140 components (defined at 100 – 180 ms post-tactile stimulus onset in each VT pair) derived in synchronous and visual-leading tactile (VT) conditions. The ED group reveals more dispersed activity in the fronto-central electrodes compared to the NH group, particularly for the synchronous and + 30 SOAs (see left two panels in Figure 4). Global dissimilarity (DISS) was calculated to quantify the topographical similarity between ED and NH at each SOA displayed. A DISS value of 1.08 for the +30 SOA was larger than expected based on the upper 5% confidence limit of the permutation analysis. This finding suggests that the spatial topography between ED and NH was indeed heterogenous while the topographies for 0 ms, +100 and +250 SOAs appear moderately homogenous (DISSs = 0.70; 0.68; 0.71; respectively). Dissimilarity analysis to compare ED and NH spatial topographies during the tactile P200 time window (190–250 ms after onset of tactile stimulus in VT conditions) (Figure 5) revealed similar activation patterns between the groups (DISS < 0.68) for all conditions, a finding supported by our permutation analysis.

**FIGURE 6:**
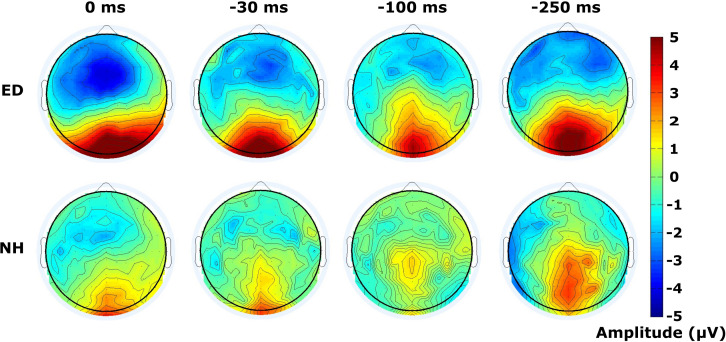
Distribution of activity across scalp within visual P100 time window. Scalp topographies of mean amplitudes within time window defining the visual P100 component are displayed for ED **(top row)** and NH **(bottom row)** groups, for synchronous and 3 tactile-leading conditions (negative SOAs).

Finally, mean amplitudes are displayed within the time window of 120–180 ms following the visual stimulus of the respective tactile-leading visual pair in Figure 6. The distribution of the positive deflection in the occipital area was observed as more widespread in ED (top row) than in NH (bottom row), particularly in the 30 ms condition as confirmed by a DISS estimate of 0.90 which surpassed our 5% confidence limit used in the permutation analysis. The other conditions induced more similar topographies between groups (DISS < 0.67).

## Discussion

Congenital or early loss of auditory input may have severe consequences for subsequent temporal detection and sensitivity. This is particularly important in understanding how perception of multisensory cues is affected, a process heavily dictated by temporal discrepancies between the sensory signals comprising the multisensory event. Visual-tactile temporal sensitivity also distinctly influences perception of body ownership and representation. For instance, susceptibility to the rubber hand illusion [when a participant feels their own hand, hidden from view, being stroked while watching a rubber hand get stroked, they feel as if the rubber hand was their own (Botvinick and Cohen, 1998; Liu and Medina, 2017)] can be predicted from a subject’s temporal sensitivity to visual-tactile asynchronies (Costantini et al., 2016). Not only is visual-tactile temporal acuity important for perceived body representation, improved development of sensory substitution devices relies on understanding the affected person’s perceptual experience, specifically what affects perception of multimodal synchrony (Kristjánsson et al., 2016). While majority of prior studies examining multisensory processing in ED have primarily relied on simultaneous stimulus presentation, the aim of the current study was to understand how early deafness affected the processing of synchronous as well as asynchronous multisensory signals.

There was no significant difference between ED and NH adults in behavioral performance accuracy, visual-tactile temporal order discrimination sensitivity or perceived visual-tactile synchrony. When the visuo-tactile pair was simultaneous, the ED group had larger amplitudes for early visual (P100) (in occipital electrodes) and early tactile (N140) (in FC electrodes) components. When the two signals were temporally offset from each other, ED had larger amplitudes of the early N140 tactile component within FC ROI for the smallest SOA conditions (± 30 ms) while ED had larger visual P100 amplitudes in occipital ROI across SOA conditions. In addition, ED showed shorter latencies of the tactile N140 component for −250 and −100 SOAs while they demonstrated significantly longer latencies for tactile P200 component across SOA levels. Finally, regardless of group, there was a similar dependence on SOA level for amplitude modulation within all ROIs examined.

The absence of any group differences in behavioral measures was somewhat surprising given prior studies that have shown impaired unisensory temporal order sensitivities (Heming and Brown, 2005; Bolognini et al., 2011) and reduced behavioral gains to multisensory versus unisensory stimuli presentation (Nava et al., 2014; Hauthal et al., 2015). However, findings from the current study as well as a prior study also reporting absence of group differences in sensitivities for discriminating visual temporal order suggest that auditory experience may not be critical for establishing a framework that allows precise discrimination of temporal order across modalities as previously described by the auditory scaffolding hypothesis (Conway et al., 2009). Follow-up studies that incorporate more levels of SOAs as well as additional temporal discrimination tasks, such as cross-modal duration perception (i.e., gap detection), would provide additional evidence that may reveal differences in multisensory temporal order perception and other temporal perceptual abilities in ED.

Despite comparable temporal order perceptual abilities, differences in amplitudes for both visual and tactile components between ED and NH during simultaneous visual-tactile stimulation reveals altered sensory processing due to auditory deprivation. There was a greater amplitude of the visual P100 component in occipital region of ED for the synchronous condition. This finding may be indicative of increased cortical resources dedicated to processing visual information or altered visual processing at early stages in ED. Heightened visual P100 amplitudes in ED measured during a visual detection task predicted reaction times suggesting enhanced unisensory processing in ED (Bottari et al., 2011). Larger amplitudes of early visual components (P110) in ED were also described by Hauthal et al. (2013) in the context of unisensory visual stimulation via alternating checkerboard patterns. One contributing factor offered as an interpretation was recruitment of posterior parietal cortex by ED, either for additional processing of or increased attention toward the visual stimuli (Hauthal et al., 2013). Similarly, while the larger visual P100 amplitudes reported in the present study do not necessarily reveal enhanced processing of the visual stimulus, it is clearly indicative of altered early visual processing in ED during bimodal stimulation.

Early processing of the tactile stimulus showed alterations in FC ROI of ED with greater tactile N140 amplitudes at the shortest SOAs tested (−30, 0, +30 ms). Increased responsiveness in somatosensory electrodes is in line with a previous study that suggested increased cortical excitability in ED for somatosensation (Güdücü et al., 2019), perhaps resulting in enhanced haptic decoding within somatosensory areas. This explanation may also help explain the larger amplitudes of ED only at the most ambiguous SOAs, conditions where greater resources would be necessary to discern the correct temporal order. While electrodes within FC region were selected to investigate somatosensory processing, this area has also previously shown reliable and comparable auditory ERPs across groups (Ponton et al., 2000; Bishop et al., 2007; Setti et al., 2011; Mahajan and McArthur, 2012; Basharat et al., 2018). Therefore, there is likely recruitment of auditory areas by ED for early stages (reflected by N140) of tactile processing, similar to the cross-modal recruitment of auditory cortex by ED for processing vibrotactile (Levänen and Hamdorf, 2001; Auer et al., 2007), visual motion (Finney et al., 2001) and visual rhythm stimuli (Bola et al., 2017). However, without source localization it is difficult to pinpoint the cortical areas leading to the enhanced response found in FC electrodes.

As multisensory integration is thought to occur in early stages of sensory processing within traditionally unisensory areas (Kayser et al., 2005; Schroeder and Foxe, 2005) as well as multimodal areas (Senkowski et al., 2008; Hauthal et al., 2013), both primary and secondary somatosensory regions, auditory areas and multimodal areas along parieto-temporal region could have contributed to the greater N140 amplitudes found in ED. Such an increase in signal strength by additional activated areas would indeed be reflected in larger amplitudes (McDonald et al., 2005). While no group differences of the later P200 tactile component amplitudes were found, ED adults did demonstrate later latencies for this later tactile component. The tactile P200 normally reflects attentional enhancement during sensory processing (Freunberger et al., 2007) and audio-tactile interactions in NH adults (Zumer et al., 2019). Taken together, these findings implicate that signal strength is not affected while speed of later tactile processing is affected by auditory deprivation.

ED adults also had significantly larger amplitudes of the early visual P100 component, regardless of SOA, within occipital ROI. This is likely consequent of cross-modal reorganization dynamics and the resulting increased influence of the tactile modality on visual as a result of early auditory deprivation (Karns et al., 2012; Hauthal et al., 2015). Indeed, tactile modulation of primary visual areas may be due to increased connectivity from somatosensory onto visual networks, as shown in early deaf cats (Stolzberg et al., 2018). Similarly, increased tactile N140 amplitudes from FC electrodes during visual-leading tactile conditions could be due to increased afferent projections from visual and somatosensory areas onto auditory areas (Wong et al., 2015) reflecting a larger amount of cortical resources dedicated to processing tactile stimuli. The widespread distribution of activity visible on the ED scalp topographies across frontal and central electrodes during tactile processing and across occipital electrodes during visual processing also suggests recruitment of additional areas and/or neuronal populations for processing visual-tactile information. However, considering the comparable behavioral performance and sensitivities, modulation of visual-tactile processing is not necessarily indicative of enhanced processing, simply altered and more distributed processing. Additionally, a prior investigation on audio-visual temporal order perception in NH adults theorized that increased amplitudes of early sensory components led to enhanced signal strength associated with the external signal evoking that component and subsequent perceptual bias toward the perceived temporal order of that signal (McDonald et al., 2005). However, the current study shows enhanced signal strength in ED for visual P100 components from all SOA levels and for tactile N140 components at the shortest SOA levels without improved behavioral performance. Therefore, we propose that in the case of early deafness, increased amplitudes and thus signal strength reflect enhanced recruitment of cortical areas to process the stimuli without any temporal primacy effect resulting in similar performance accuracy and sensitivity across groups.

Compensatory mechanisms, such as increased cortical activation, may be largely driven by the haptic modality which is consistent with modality appropriateness, a hypothesis proposing that the sensory modality with greater resolution for the task at hand exerts greater influence in the subsequent processing and perception of the multisensory event. As the tactile modality has a heightened temporal resolution compared to the visual domain (Baumgarten et al., 2017), the tactile cues should be given greater perceptual weight during the present TOJ task. For NH adults, auditory information dominates temporal processing (Walker and Scott, 1981; Welch et al., 1986), however, under absence of audition (i.e., deafness) tactile information becomes the most reliable modality for temporal processing. If the ED group does indeed more heavily weigh tactile information for temporal processing, this could be reflected in the subsequent influence on visual areas. For instance, when tactile preceded visual information, the leading tactile stimulus was likely more salient in ED increasing the reliability and detection acuity needed to perceive temporal order. The earlier latencies found for ED in the −250 and −100 ms (tactile-leading) SOA conditions implies faster processing of the tactile stimulus by ED when there is reduced influence from a visual stimulus, possibly enhancing the saliency of the tactile cue. A similar finding for visual saliency and faster visual processing was reported in ED performing a spatial task, a domain dominated by the visual modality (Heimler et al., 2017). Follow up studies directly manipulating the reliability of visual and tactile signals are necessary to fully understand how saliency of one modality influences processing of the second modality in a temporal order discrimination task. In addition, source localization is required to discern the cortical sources producing these responses measured in FC and occipital electrodes to more fully understand what regions are directly modulated by tactile and visual systems.

One common finding for both groups was the amplitude modulation of tactile components dependent on the SOA. For tactile-leading visual conditions within occipital ROI, the amplitude of the early P100 component was largest for 30 ms condition compared to 100 ms and 250 ms SOA conditions. In a similar study, early sensory processing components of the lagging stimulus in an audio-visual pair showed reduced amplitudes in older versus young adults at the large SOA (270 ms) but not small SOA (70 ms) (Setti et al., 2011). In conjunction with the reduced precision of older adults performing a TOJ task, the authors hypothesized that the lower amplitudes reflect reduced processing of the second signal and integration of the 2 cues at this large delay (Setti et al., 2011). However, a study replicating the design of Setti et al. (2011) showed opposing results (reduced amplitude for young compared to older at the same large SOA – 270 ms) (Basharat et al., 2018). This was interpreted as a reduced ability for older individuals to disengage their attention from the second stimulus as compared to young. In the present study, the reduction of early tactile N140 and early visual P100 amplitudes with increasing SOA was present in both groups. In line with the interpretation of Basharat et al. (2018), this could indicate a reallocation or reduction of cognitive resources in processing the secondary tactile stimulus presented at a later delay for all participants. As behavioral performance also increased with increasing SOA, the larger delay likely improved perceptual resolution to discern temporal order and dedicated processing of the second stimulus wasn’t required.

Results presented from this study showcase some alterations to processing visual-tactile stimuli between ED and NH participants. ED adults had larger amplitudes for early visual and tactile processing components estimated from the simultaneous visual-tactile condition suggesting increased cognitive resources for multisensory processing after early auditory deprivation. In addition, ED adults had larger tactile N140 components within FC electrodes at the shortest SOAs (± 30 ms). These findings along with the broader activation patterns observed on the scalp topographies of ED during the early time window post-tactile onset suggest compensatory mechanisms and potential recruitment of auditory areas by ED to process tactile information but not enhanced temporal processing. Future studies probing additional visual-tactile tasks (i.e., detection or spatial tasks) would further determine if cortical processing differences in ED, as reported in our study, are common across global visual-tactile processing or specific to temporal processing. Finally, ED adults also had larger visual P100 components estimated from occipital electrodes for all SOA conditions likely due to cross-modal reorganization of tactile inputs onto visual areas as well as modality appropriateness of the tactile system for temporal processing tasks.

## Data Availability Statement

The de-identified raw data supporting the conclusions of this article will be made available by the authors upon request.

## Ethics Statement

The studies involving human participants were reviewed and approved by Institutional Review Board at the University of Nevada, Reno. The participants provided their written informed consent to participate in this study.

## Author Contributions

AS and FJ designed the experiment and interpreted the data. AS and KC acquired the data and conducted statistical analysis. AS wrote the manuscript. FJ critically evaluated the manuscript. All authors contributed to the article and approved the submitted version.

## Conflict of Interest

The authors declare that the research was conducted in the absence of any commercial or financial relationships that could be construed as a potential conflict of interest.
