# Supplementary material for: Electrophysiological Dynamics of Visual-Tactile Temporal Order Perception in Early Deaf Adults
Source: Front Neurosci. 2020 Sep 23;14:544472. doi: 10.3389/fnins.2020.544472 (PMC7539666; doi:10.3389/fnins.2020.544472)
Supplement: Supplementary file 1 [file Table_1.docx]

**Supplementary Material – Table 1**

Electrophysiological Dynamics of Visual-Tactile Temporal Order Perception in Early Deaf Adults

Alexandra N. Scurry^1*^, Kudzai Chifamba^1^, Fang Jiang^1^

^1^Department of Psychology, University of Nevada, Reno, Nevada, USA

*** Correspondence:**Alexandra N. Scurry
[ascurry@unr.edu](mailto:ascurry@unr.edu)

**Supplementary Table 1.** Group averages and standard errors (in parenthesis) of tactile N140 amplitudes and latencies in Fronto-Central (FC) ROI.

|  | **Amplitude (µV)** | |  | **Latency (ms)** | |
| --- | --- | --- | --- | --- | --- |
| **SOA** | **ED** | **NH** |  | **ED** | **NH** |
| **-250** | -2.88 (.46) | -2.06 (.37) |  | 148.76 (2.68) | 159.99 (3.47) |
| **-100** | -2.92 (.43) | -2.20 (.37) |  | 147.46 (2.90) | 157.88 (3.50) |
| **-30** | -4.80 (.39) | -2.29 (.42) |  | 151.04 (2.93) | 154.95 (2.89) |
| **0** | -5.03 (.63) | -2.22 (.49) |  | 144.69 (3.76) | 151.37 (4.78) |
| **+30** | -2.89 (.30) | -0.85 (.44) |  | 126.90 (7.05) | 135.20 (9.83) |
| **+100** | -1.62 (.54) | -0.28 (.61) |  | 151.30 (7.66) | 133.89 (7.26) |
| **+250** | -2.14 (.66) | -0.60 (.56) |  | 147.95 (9.12) | 126.63 (8.51) |
