# Supplementary material for: Electrophysiological Dynamics of Visual-Tactile Temporal Order Perception in Early Deaf Adults
Source: Front Neurosci. 2020 Sep 23;14:544472. doi: 10.3389/fnins.2020.544472 (PMC7539666; doi:10.3389/fnins.2020.544472)
Supplement: Supplementary file 2 [file Table_2.docx]

**Supplementary Material – Table 2**

Electrophysiological Dynamics of Visual-Tactile Temporal Order Perception in Early Deaf Adults

Alexandra N. Scurry^1*^, Kudzai Chifamba^1^, Fang Jiang^1^

^1^Department of Psychology, University of Nevada, Reno, Nevada, USA

*** Correspondence:**Alexandra N. Scurry
[ascurry@unr.edu](mailto:ascurry@unr.edu)

**Supplementary Table 2.** Group averages and standard errors (in parenthesis) of tactile P200 amplitudes and latencies in Fronto-Central (FC) ROI.

|  | **Amplitude (µV)** | |  | **Latency (ms)** | |
| --- | --- | --- | --- | --- | --- |
| **SOA** | **ED** | **NH** |  | **ED** | **NH** |
| **-250** | 2.36 (.48) | 1.15 (.70) |  | 233.24 (3.87) | 220.38 (3.41) |
| **-100** | 0.90 (.34) | 0.89 (.61) |  | 219.56 (6.37) | 220.21 (5.99) |
| **-30** | 4.26 (.50) | 2.65 (.68) |  | 236.49 (4.66) | 220.87 (5.05) |
| **0** | 4.17 (.49) | 2.66 (.63) |  | 227.54 (4.66) | 222.01 (4.61) |
| **+30** | 1.40 (.57) | 0.98 (.68) |  | 227.98 (5.00) | 227.81 (4.04) |
| **+100** | 0.67 (.62) | 0.03 (.55) |  | 232.68 (5.75) | 223.24 (6.99) |
| **+250** | 2.04 (.74) | 1.91 (.86) |  | 240.89 (2.96) | 240.23 (4.85) |
