# Supplementary material for: Electrophysiological Dynamics of Visual-Tactile Temporal Order Perception in Early Deaf Adults
Source: Front Neurosci. 2020 Sep 23;14:544472. doi: 10.3389/fnins.2020.544472 (PMC7539666; doi:10.3389/fnins.2020.544472)
Supplement: Supplementary file 3 [file Table_3.docx]

**Supplementary Material – Table 3**

Electrophysiological Dynamics of Visual-Tactile Temporal Order Perception in Early Deaf Adults

Alexandra N. Scurry^1*^, Kudzai Chifamba^1^, Fang Jiang^1^

^1^Department of Psychology, University of Nevada, Reno, Nevada, USA

*** Correspondence:**Alexandra N. Scurry
[ascurry@unr.edu](mailto:ascurry@unr.edu)

**Supplementary Table 3.** Group averages and standard errors (in parenthesis) of visual P100 amplitudes and latencies in occipital ROI.

|  | **Amplitude (µV)** | |  | **Latency (ms)** | |
| --- | --- | --- | --- | --- | --- |
| **SOA** | **ED** | **NH** |  | **ED** | **NH** |
| **-250** | 5.29 (1.01) | 2.49 (.69) |  | 138.07 (3.92) | 155.36 (5.64) |
| **-100** | 4.62 (.64) | 2.14 (.64) |  | 145.49 (5.71) | 148.93 (5.03) |
| **-30** | 7.81 (.66) | 4.10 (.64) |  | 135.26 (4.29) | 136.19 (5.15) |
| **0** | 7.85 (.96) | 4.29 (.73) |  | 153.85 (3.63) | 155.89 (5.12) |
| **+30** | 6.15 (.81) | 3.42 (.63) |  | 144.68 (6.01) | 142.40 (6.84) |
| **+100** | 5.67 (.79) | 2.72 (.71) |  | 145.73 (5.39) | 148.44 (6.40) |
| **+250** | 5.48 (.89) | 2.92 (.71) |  | 145.43 (5.10) | 145.42 (6.03) |
